# Field evaluation of nanopore targeted next-generation sequencing to predict drug-resistant tuberculosis from native sputum in South Africa and Zambia

**DOI:** 10.1128/jcm.01390-24

**Published:** 2025-02-12

**Authors:** Tiana C. Schwab, Lavania Joseph, Andrew Moono, Pauline C. Göller, Mamello Motsei, Guy Muula, Denise Evans, Stefan Neuenschwander, Gunar Günther, Carolyn Bolton, Peter M. Keller, Alban Ramette, Matthias Egger, Shaheed V. Omar, Lukas Fenner, on behalf of IeDEA Southern Africa (IeDEA-SA)

**Affiliations:** 1 Institute of Social and Preventive Medicine, University of Bern 30317 , Bern, Switzerland; 2 Centre for Tuberculosis, National & WHO Supranational TB Reference Laboratory, a division of the National Health Laboratory Services, National Institute for Communicable Diseases 70687 , Johannesburg, South Africa; 3 Center for Infectious Disease Research in Zambia 248489 , Lusaka, Zambia; 4 Institute of Medical Microbiology, University of Zürich 27217 , Zürich, Switzerland; 5 Health Economics and Epidemiology Research Office, Faculty of Health Sciences, University of the Witwatersrand 37708 , Johannesburg, South Africa; 6 Institute for Infectious Diseases, University of Bern Institute for Infectious Diseases 87618 , Bern, Switzerland; 7 Department of Pulmonology and Allergology, Inselspital Universitatsspital Bern 27252 , Bern, Switzerland; 8 Department of Medical Science, Faculty of Health Sciences, University of Namibia 99404 , Windhoek, Namibia; 9 Clinical Bacteriology/Mycology, University Hospital Basel 30262 , Basel, Switzerland; 10 Centre for Infectious Disease Epidemiology & Research, School of Public Health & Family Medicine, University of Cape Town 37716 , Cape Town, South Africa; 11 Population Health Sciences, University of Bristol 152358 , Bristol, United Kingdom; University of Manitoba, Winnipeg, Manitoba, Canada

**Keywords:** tuberculosis, implementation, next-generation sequencing, nanopore sequencing, resistance prediction, low- and middle-income countries

## Abstract

Rapid and comprehensive drug susceptibility testing (DST) is essential for diagnosing and treating drug-resistant tuberculosis effectively, and next-generation sequencing can be an effective genotypic DST method. We implemented and evaluated the performance of a nanopore targeted sequencing assay, called the Tuberculosis Drug Resistance Test (TBDR, Oxford Nanopore Diagnostics, Ltd., United Kingdom), which predicts drug resistance to 16 TB drugs, at a South African reference laboratory and a district diagnostic laboratory in Zambia. We compared the sequencing success rates between unprocessed and decontaminated sputum samples and determined the diagnostic accuracy against local DST (Xpert MTB/RIF Ultra, Xpert MTB/XDR, and BD BACTEC MGIT phenotypic DST). We prospectively sequenced 236 samples and have 148 samples with sequencing results from unprocessed and decontaminated sputum. We obtained successful sequencing results from 66.4% (94/148) unprocessed sputum samples and 75% (111/148) decontaminated samples. Sequencing success rates at the two sites differed, with 50.7% (36/71) successful sequencing results from unprocessed sputum in Zambia and 75.3% (58/77) in South Africa. Samples with “low” bacterial load, measured by Xpert MTB/RIF Ultra, tended to produce fewer successful sequencing results. TBDR sequencing predicted resistances in 48 samples, detecting resistance for rifampicin (*n* = 41) and isoniazid (*n* = 20), as well as 10 second-line drugs (*n* = 15). Sensitivity was variable compared to phenotypic DST, ranging from 33 (ethionamide) to 94% (rifampicin), while specificity remained above 90% for all drugs, except clofazimine. The TBDR assay can provide rapid, comprehensive genotypic DST. Technical and operational challenges need to be addressed for its broader implementation in high tuberculosis-burden settings.

**IMPORTANCE:**

This study illustrates the use of the Tuberculosis Drug Resistance Test (TBDR, Oxford Nanopore Diagnostics, Ltd., United Kingdom) as a rapid drug susceptibility testing (DST) approach for diagnosing drug-resistant TB in the high TB-burden countries of South Africa and Zambia. The TBDR assay predicts resistance to 16 TB drugs, including first- and second-line treatments. By implementing the TBDR assay in a national reference laboratory in South Africa and a district diagnostic laboratory in Zambia, we demonstrate how this technology can provide faster diagnostic results (days) compared to traditional phenotypic DST methods (~2 months), with adequate sensitivity. Missed resistances compared to phenotypic DST indicate that technical improvements are needed. Successful sequencing from unprocessed and decontaminated sputum samples at different sites suggests feasibility in diverse settings, though operational challenges remain. Implementing this rapid, comprehensive DST approach could enhance drug-resistant tuberculosis diagnosis and treatment, ultimately improving patient outcomes and helping to combat tuberculosis in high-burden regions.

## INTRODUCTION

There is a large diagnostic gap for drug-resistant tuberculosis (TB). Of an estimated 62,000 incident drug-resistant TB cases in Africa in 2022, only 22,495 (36%) were diagnosed (1). A lack of rapid drug susceptibility testing (DST), particularly for new TB drugs, such as bedaquiline and pretomanid, severely limits the timely detection of resistance and impedes the effectiveness of new short-course treatments for drug-resistant TB. Despite the availability of some form of second-line DST in 28 of 47 African countries, testing of notified rifampicin-resistant cases remains limited (2). DST testing for new and repurposed drugs is not available or introduced long after the drugs become available for clinical use. The current reference standard for DST, phenotypic culture-based DST, takes several weeks and requires biosafety level-three laboratory infrastructure, which is unavailable in many settings. Genotypic DST methods detect mutations that are associated with drug resistance, and commercial nucleic acid amplification tests are fast and can be simple to use. Tests, such as the GenoType MTBDRsl (Hain Life Sciences, Nehren, Germany) and Xpert MTB/XDR (Cepheid, Sunnyvale, CA, USA), detect resistances to second-line TB drugs, including fluoroquinolones and aminoglycosides, but remain restricted to a limited set of drugs.

Targeted next-generation sequencing (tNGS) offers an attractive addition to current DST methods. By combining high-throughput sequencing with DNA amplification of multiple gene targets associated with resistance, it can simultaneously predict resistance to a wide range of TB drugs (3–7). The World Health Organization (WHO) has recently endorsed tNGS as a follow-on diagnostic test for detecting drug resistance to specific drugs (8) and in various settings tNGS assays have shown a high diagnostic accuracy for several drugs (8, 9). However, most of the studies conducted in sub-Saharan Africa sequenced samples in North America or Europe (9), and there are limited published data on the use of tNGS in routine practice, especially in lower-income, high-burden TB settings. And while tNGS can be performed directly on clinical samples (6, 10, 11), which is advantageous for routine testing, preprocessing may affect the performance of tNGS and requires further investigation.

This study evaluated nanopore tNGS in diagnosing drug-resistant TB in South Africa and Zambia. We implemented the Tuberculosis Drug Resistance Test (TBDR, Oxford Nanopore Diagnostics, Ltd., United Kingdom), which predicts drug resistance for 16 TB drugs, in Johannesburg, South Africa and Lusaka, Zambia. We prospectively field-tested the assay in sputum in two TB cohorts, evaluated the sequencing success rates, compared results between unprocessed (native) and decontaminated sputum samples, and assessed the diagnostic accuracy of the TBDR assay against locally available DST.

## MATERIALS AND METHODS

### Study design, setting, participants, and study procedures

We conducted a prospective field evaluation and a diagnostic accuracy study of the TBDR assay in sputum samples from persons with microbiologically confirmed active pulmonary TB. We collected samples from individuals in Johannesburg, South Africa and Lusaka, Zambia, who are participating in an ongoing tuberculosis cohort study (12), with additional recruitment of drug-resistant TB patients from Sizwe Tropical Diseases Hospital in South Africa and the Copper Belt region in Zambia. Details about the two study sites are provided in Table 1. Participants were recruited from October 2022 to March 2024. Trained health workers collected demographic and clinical data using standardized forms (12). TB diagnosis and treatment were managed according to local standard of care.

**TABLE 1:** Tuberculosis incidence, recruitment sites, and laboratory capacities at the study sites^*a*^

	South Africa	Zambia
Total TB incidence, rate per 100,000 population (range)^*b*^	468 (304–665)	295 (184–431)
MDR/RR-TB incidence, rate per 100,000 population (range)^*b*^	19 (11–26)	9.7 (4.9–15)
TB treatment	According to the national TB program	According to the national TB program
MDR treatment	6-month BPaL and BPaL-L	WHO-recommended treatment regimen
Sites		
	*City of Johannesburg* Themba Letu Clinic Sizwe Tropical Diseases Hospital	*Greater Lusaka area* Chawama First Level Hospital Kanyama First Level Hospital *Copper Belt region* Ndola and Kitwe Teaching Hospitals Rona Thompson and Nchanga North General Hospital
Laboratories		
Name	National Health Laboratory Services (NHLS) and National Institute for Communicable Diseases (NICD)—Center for Tuberculosis	Center for Infectious Disease Epidemiology and Research in Zambia (CIDRZ) Laboratory
Type of laboratory	National and WHO supranational TB reference laboratory	Non-governmental, district diagnostic laboratory
Previous NGS experience	Yes	No

^*a*^ TB, tuberculosis; MDR—multidrug-resistant TB; RR—rifampicin-resistant TB; NGS—next-generation sequencing.

^*b*^ World Health Organization (2023) Global Tuberculosis Report 2023. Geneva, Switzerland.

We included samples with sufficient sputum volume (>2 mL) from individuals aged 15 years and older who tested positive for TB with a semi-quantitative Xpert MTB/RIF Ultra result of “low” or higher. We consecutively sequenced eligible samples in batches as participants were recruited. Given the low number of drug-resistant cases in the cohort, we additionally sequenced 20 decontaminated sputum samples with drug resistance to second-line drugs available from routine drug resistance surveillance in South Africa.

### Laboratory procedures

Sputum samples were homogenized by pipetting and aliquoted for Xpert MTB/RIF Ultra, TBDR sequencing, and decontamination. We used unprocessed sputum for Xpert MTB/RIF Ultra testing. For TBDR sequencing, we inactivated an unprocessed sputum aliquot for 30 min at 96°C (hereafter referred to as unprocessed sample). The remaining sample was decontaminated using the standard N-acetyl-L cysteine–sodium hydroxide (NALC–NaOH) method (13) with a final NaOH concentration of 1.5%, retaining the concentrated sediment for TBDR sequencing (decontaminated sample), phenotypic DST, and Xpert MTB/XDR.

#### Index test

We homogenized the inactivated or decontaminated sputum samples by bead-beating using a FastPrep-24 5G (MP Biomedicals, Irvine, CA, USA) and extracted DNA using the Maxwell RSC Blood DNA Kit on a Promega Maxwell RSC (Promega, Madison, WI, USA). We amplified gene targets using the PCR and library preparation methods described in the Oxford Nanopore Diagnostics Tuberculosis Drug Resistance Test (TBDR) protocol. The TBDR assay predicts drug resistance for 16 TB drugs by amplifying 24 gene regions associated with drug resistance (DR targets) by multiplex PCR. Each drug is associated with one to five gene targets, and some targets contain variants that confer cross-resistance to multiple drugs within a class (Table S1). The TBDR assay also amplifies the *hsp65* region for Mycobacteria speciation and a spoligotyping target for typing *Mycobacterium tuberculosis* complex (MTBC) strains. We used the Rapid Barcoding Kit 96 (SQK-RBK110.96, Oxford Nanopore Technologies, United Kingdom) to barcode up to 22 samples and two controls per sequencing run and sequenced libraries using MinION R9.4.1 flow cells for 2 h. We analyzed the sequencing data using the EPI2ME wf-tb-amr v2.0.0-alpha.4 workflow, which identifies variants associated with antibiotic resistance based on the WHO Catalogue of Mutations in *Mycobacterium tuberculosis* complex*,* 1st edition (2021) (14) and an internal catalogue by Oxford Nanopore Technologies, which is not publicly available.

Using the default parameters of the wf-tb-amr workflow, sequencing of a sample was considered successful if 15 or more DR targets, the *hsp65* target, or the internal control passed the coverage threshold (median coverage of 20× or higher). If less than 15 DR targets passed the coverage threshold, drug susceptibility predictions were not reported. For each drug, a sample was considered resistant to a drug if a variant associated with drug resistance was detected with an allele frequency of at least 15% in any associated gene targets. It was considered susceptible to a drug if the target(s) passed the coverage threshold, and no associated variants were detected and undetermined if one or more of the associated targets had insufficient coverage (<20× median coverage) for variant calling. A sequencing run was considered successful if the no-template control had less than 20× median coverage in fewer than three targets, and 22 or more targets of the positive control had a median coverage of 20× or higher. If either the no-template or positive control failed, all test samples failed, and no results were reported (Table S2).

#### Reference tests

We used locally available DST as reference standards. At both sites, we tested for rifampicin resistance using Xpert MTB/RIF Ultra and performed phenotypic DST using the BD BACTEC Mycobacterial Growth Indicator Tube 960 System (BACTEC MGIT 960, Becton Dickinson, Sparks, MD, USA) for rifampicin (0.5 µg/mL), isoniazid, ethambutol, and pyrazinamide at WHO-recommended critical concentrations (Table S1). In South Africa, we additionally tested decontaminated samples with the Xpert MTB/XDR (Cepheid, Sunnyvale, CA, USA) to detect mutations associated with resistance to isoniazid, fluoroquinolones, second-line injectable drugs (amikacin, kanamycin, capreomycin), and ethionamide (Table S1). For the supplementary drug-resistant samples, BACTEC MGIT 960 phenotypic DST was done per manufacturer’s recommendations using WHO-recommended critical concentrations (15, 16) for rifampicin, isoniazid, pyrazinamide, streptomycin, amikacin, kanamycin, capreomycin, ethionamide, levofloxacin, moxifloxacin, linezolid, bedaquiline, and clofazimine. For ethambutol and delamanid, mutations classified as resistance-conferring by the WHO Catalogue of Mutations, 2nd edition (2023) (17) were detected using Illumina NextSeq 2000 (Illumina, San Diego, CA, USA) whole-genome sequencing.

### Implementation and validation of the TBDR assay

We conducted in-depth training with the study and laboratory teams on the study procedures and the index test at the beginning of the study (1 week in South Africa, 2 weeks in Zambia), with two refresher training sessions during the study in Zambia. The training covered study standard operating procedures on sputum collection and laboratory procedures, including DNA extraction, library preparation, NGS, and bioinformatics. We held regular online meetings throughout the study to discuss progress and troubleshoot technical issues, and laboratory teams provided feedback on the quality and quantity of the sputum collected.

To verify the reproducibility of TBDR assay outcomes in a subsample, we re-sequenced the DNA extracted from 20 paired unprocessed and decontaminated samples (*n* = 40) using the TBDR protocol at a central laboratory (Institute for Infectious Diseases, University of Bern, Switzerland). We randomly selected samples, balancing sampling to ensure that all paired outcome combinations are represented and selecting 12 samples from Zambia and eight from South Africa.

### Outcomes and analysis

To assess the performance of the TBDR assay, we compared sequencing success rates and fitted a mixed-effects logistic regression model to the data, including fixed effects for bacterial load, site and decontamination, and random effects to account for the matching of the samples. Results are presented as odds ratios (OR) with 95% confidence intervals (95% CI).

We determined the accuracy of the TBDR assay by calculating sensitivity and specificity with 95% CI using locally available DST as the reference standard. Sensitivity was the proportion of samples classified as drug-resistant by the reference standard for which TBDR detected a resistance-conferring variant. Specificity was the proportion of samples identified as drug-susceptible by the reference test where no resistance-conferring variant was detected by tNGS. The sample was excluded from the accuracy calculations for a drug target if no reference test or sequencing result was available for that drug or if sequencing was unsuccessful for all corresponding drug targets. To calculate a total test accuracy of sample-drug combinations, we summed counts of true positive, false positive, false negative, and true positive calls across all drugs. Sequencing results from individual runs were combined using custom Python scripts, and analyses were conducted using R version 4.3.1.

### Reporting statement

We aligned the reporting of the study to the Standards for Reporting Diagnostic Accuracy studies (18) (Table S3).

## RESULTS

**Fig 1:**
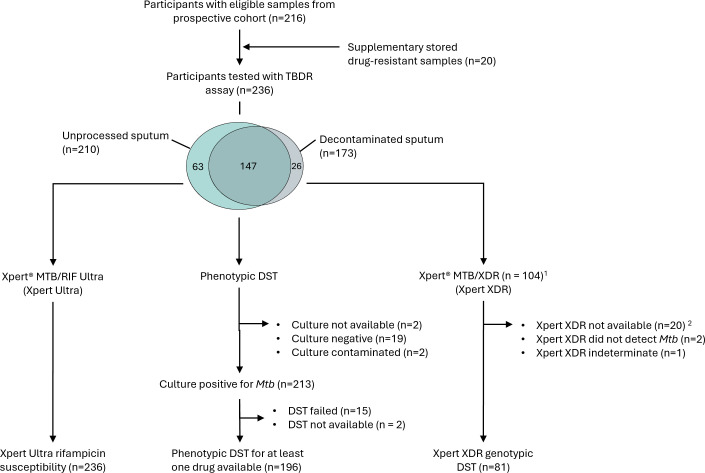
Flowchart of sputum samples processed for TBDR sequencing using unprocessed and/or decontaminated sputum, Xpert MTB/RIF Ultra, phenotypic drug susceptibility testing (DST), and Xpert MTB/XDR. Mtb—Mycobacterium tuberculosis.^1^Xpert MTB/XDR only available in South Africa. ^2^Xpert MTB/RIF Ultra and Xpert MTB/XDR not available for 20 supplementary drug-resistant samples with culture-confirmed *Mtb*.

We tested 236 participants with the TBDR assay: 132 participants in Zambia and 104 in South Africa. Table 2 describes the demographic and clinical characteristics of the participants. For 147 participants, both the unprocessed and decontaminated sputum were available for sequencing. The sites shared TBDR results, including predicted drug resistances within 24 h of DNA extraction, with the number samples per sequencing run ranging from 5 to 22. Rifampicin susceptibility results from Xpert MTB/RIF Ultra were available for all samples; phenotypic DST results were available for 186/236 samples, and genotypic DST results from the Xpert MTB/XDR (available only in South Africa) were available for 81/104 (77.9%) samples (Fig. 1; Table 3). Spoligotypes determined by TBDR sequencing were obtained from 52/211 (24.6%) unprocessed samples and 90/173 (52%) decontaminated samples. The most prevalent spoligotype detected from decontaminated samples in Zambia was the T family (Lineage 4), while in South Africa, the Beijing spoligotype (Lineage 2) was the most prevalent (Table S4).

**TABLE 2:** Demographic and clinical patient characteristics of included study samples, overall and by country^*b*^

	Overall (*n* = 236)	South Africa (*n* = 104)	Zambia (*n* = 132)
Median age, years (IQR)^*a*^	35 (29–46)	37 (30–47)	33 (29–43)
Sex at birth, n (%)			
Female	60 (25)	32 (31)	28 (21)
Male	176 (75)	72 (69)	104 (78.8)
Person living with HIV, n (%)			
Negative HIV test	119 (50)	38 (37)	81 (61)
Positive HIV test	87 (37)	36 (35)	51 (39)
Unknown	30 (13)	30 (29)	0 (0)
History of active tuberculosis, n (%)			
No	150 (64)	57 (55)	93 (70)
Yes	56 (24)	19 (18)	37 (28)
Unknown	30 (13)	28 (27)	2 (1.5)

^*a*^ Age was unknown for eight participants.

^*b*^ IQR, interquartile range.

**TABLE 3:** Number of samples with a drug susceptibility result (resistant/susceptible) per drug for each diagnostic test used^*a*^^,^^*b*^

	TBDR decontaminated	TBDR unprocessed	Xpert MTB/RIF Ultra	Phenotypic DST	WGS	Xpert MTB/XDR
Rifampicin (RIF)	103	101	236	189		
Isoniazid (INH)	97	91		188		81
Ethambutol (EMB)	100	102		168	20	
Pyrazinamide (PZA)	101	96		192		
Streptomycin (STM)	96	77		109		
Amikacin (AMK)	97	84		20		81
Capreomycin (CAP)	95	78		20		81
Kanamycin (KAN)	95	82		20		81
Ethionamide (ETH)	96	91		20		81
Levofloxacin (LFX)	103	95		20		80
Moxifloxacin (MXF)	103	95		20		
Bedaquiline (BDQ)	97	95		20		
Clofazimine (CFZ)	97	97		20		
Linezolid (LZD)	92	72		20		
Delamanid (DLM)	94	88			20	
Pretomanid (PMD)	94	88				

^*a*^ TBDR unprocessed—TBDR sequencing using unprocessed samples; TBDR decontaminated—TBDR sequencing using decontaminated samples; WGS—whole-genome sequencing.

^*b*^ For the remaining samples, the DST test failed, or the corresponding drug targets were undetermined.

### Sequencing success rates for unprocessed and decontaminated sputum

**Fig 2:**
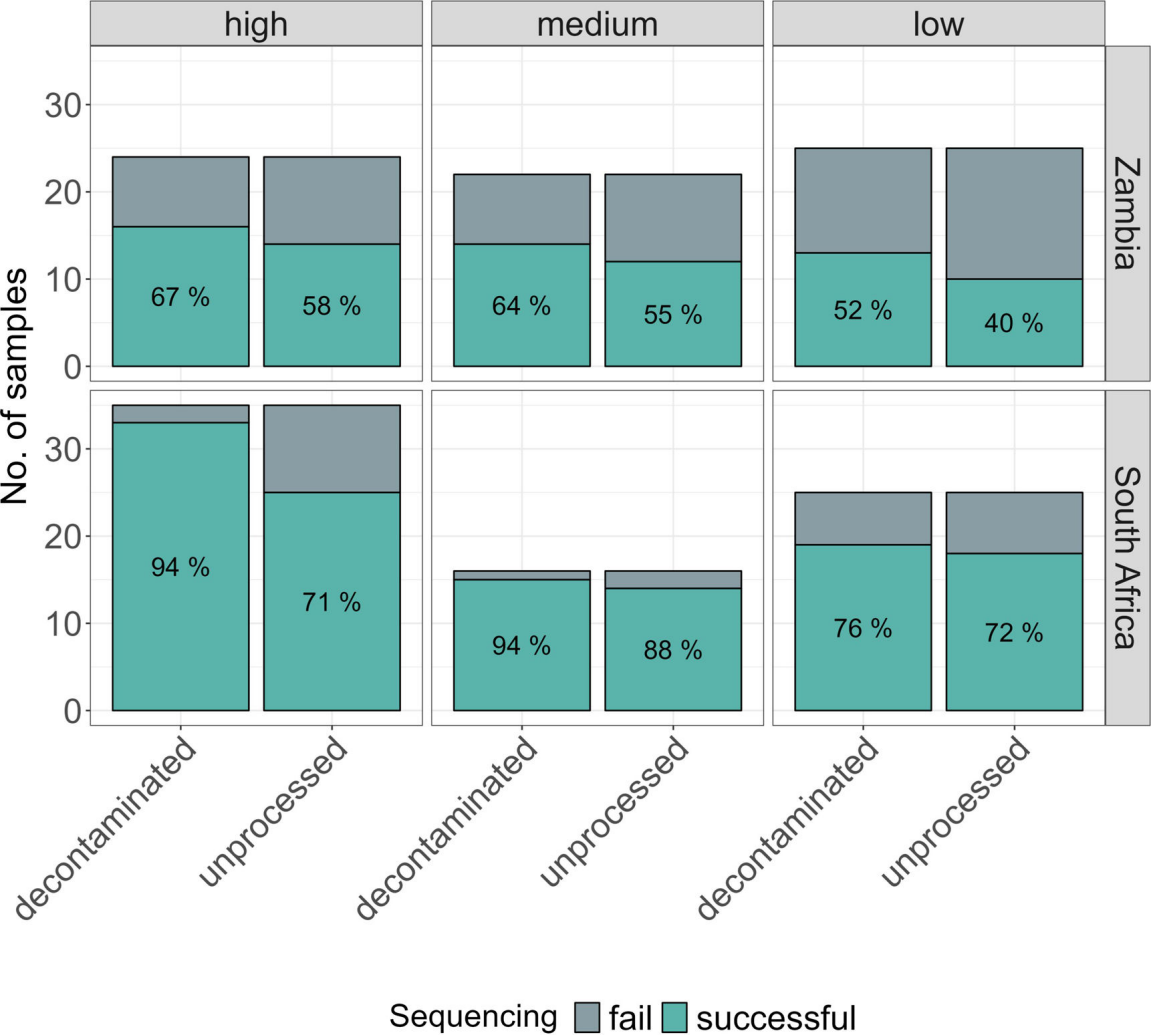
TBDR success rates when using decontaminated and unprocessed samples stratified by site and initial bacterial load, as measured by Xpert MTB/RIF Ultra from 148 participants with paired sequencing results (296 sequencing results).

We compared sequencing success between unprocessed and decontaminated samples from 147 participants with paired TBDR sequencing results from unprocessed and decontaminated sputum (76 from South Africa and 71 from Zambia). Overall, sequencing was successful in 93/147 (63.3%) unprocessed sputum samples and 110/147 (74.8%) decontaminated samples. Success rates at the two sites differed, with 36/71 (50.7%) successful sequencing results from unprocessed sputum in Zambia and 57/76 (75.0%) from South Africa. Success rates were higher with decontaminated sediments: 43/71 (60.6%) in Zambia and 67/76 (88.2%) in South Africa (Fig. 2).

In the multivariable logistic regression analysis (Table 4), successful sequencing was less likely with unprocessed samples compared to decontaminated samples (adjusted OR 0.51, 95% CI 0.29–0.90). Sequencing success was also associated with the study site, with higher success in South Africa than in Zambia (adjusted OR 4.25, 95% CI 2.16–8.35). A lower bacterial load of the sputum sample measured semi-quantitatively by Xpert MTB/RIF Ultra was associated with lower sequencing success, but associations did not reach statistical significance (*P* = 0.07).

**TABLE 4:** Variables associated with successful TBDR sequencing for 148 paired samples (296 sequencing results)

Variable	LRT^*a*^	Crude OR (95% CI)^*b*^	LRT^*c*^	Adjusted OR (95% CI)^*d*^
Xpert MTB/RIF Ultra bacterial load	*P* = 0.07		*P* = 0.07	
Low (*n* = 102)		0.46 (0.23, 0.94)		0.51 (0.25, 1.03)
Medium (*n* = 76)		0.88 (0.40, 1.90)		1.14 (0.52, 2.51)
High (*n* = 118)		1.00		1.00
Site	*P* < 0.001		*P* < 0.001	
South Africa (*n* = 154)		3.99 (2.10, 7.60)		4.25 (2.16, 8.35)
Zambia (*n* = 142)		1.00		1.00
Decontamination	*P* = 0.02		*P* = 0.02	
No—unprocessed (*n* = 148)		0.52 (0.30, 0.92)		0.51 (0.29, 0.90)
Yes—decontaminated (*n* = 148)		1.00		1.00

^*a*^ *P*-values from likelihood ratio tests (LRT) comparing the univariate model to the intercept model.

^*b*^ Crude odds ratio (OR) from logistic regression models using patient-specific random effects to account for paired data.

^*c*^ *P*-values from likelihood ratio tests comparing the log likelihoods of the multivariate model and the model, excluding the corresponding variable.

^*d*^ Adjusted OR calculated mixed-effects logistic regression model, including fixed effects for bacterial load, site and decontamination, and patient-specific random effects to account for paired data.

The percentage of successfully sequenced gene targets was higher and more consistent across gene targets for decontaminated samples, particularly in South Africa (Fig. S1). Unprocessed samples had a higher median number of undetermined targets depending on bacterial load and sequencing site (Fig. S2). The final number of samples with an interpretable drug susceptibility result (resistant/susceptible) per drug for each diagnostic test used is shown in Table 3. Considering the 384 sequencing results for all 236 participants (obtained from 210 unprocessed and 173 decontaminated samples), sequencing was successful in 143/210 (68.1%) unprocessed and 128/173 (74%) decontaminated samples.

### Predicted drug resistances by TBDR sequencing

**Fig 3:**
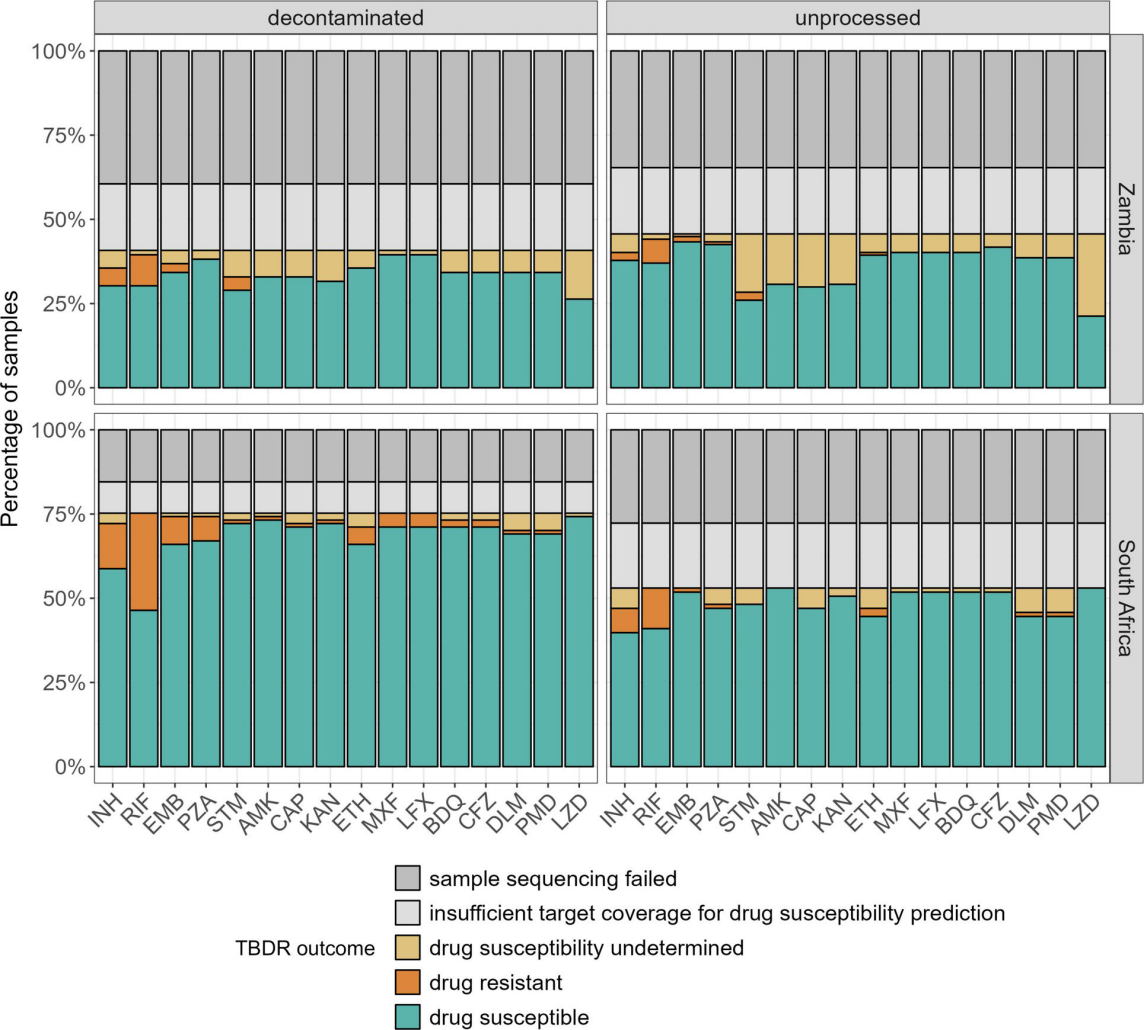
Percentage of samples with drug susceptibility predicted by TBDR sequencing, stratified by tuberculosis drug (*n* = 236). Predicted drug susceptibility was not reported for samples if fewer than 15 gene targets associated with drug resistance passed the coverage threshold (insufficient target coverage). Drug susceptibility was undetermined by TBDR sequencing for a given drug if one or more of the associated gene targets had insufficient coverage for variant calling. INH—isoniazid, RIF—rifampicin, EMB—ethambutol, PZA—pyrazinamide, STM—streptomycin, AMK—amikacin, CAP—capreomycin, KAN—kanamycin, ETH—ethionamide, MXF—moxifloxacin, LFX—levofloxacin, BDQ—bedaquiline, CFZ—clofazimine, DLM—delamanid, PMD—pretomanid, and LZD—linezolid.

Of 236 participants, TBDR sequencing identified 48 as drug resistant. We detected noticeable resistance for rifampicin (*n* = 41) and isoniazid (*n* = 20) at both sites and samples resistant to ethambutol (*n* = 11), pyrazinamide (*n* = 8), streptomycin (*n* = 6), ethionamide (*n* = 6), levofloxacin and moxifloxacin (*n* = 4), bedaquiline and clofazimine (*n* = 2), delamanid and pretomanid (*n* = 2), amikacin, kanamycin, and capreomycin (*n* = 1), (Fig. 3). The detected variants are listed in Table S5. Predicted drug susceptibilities by tNGS were 99.6% (715/718) concordant between unprocessed and decontaminated samples for which drug susceptibility results were available. Differences in variants detected in the unprocessed and decontaminated sample are listed in Table S6.

### Diagnostic accuracy of TBDR sequencing compared to local DST

**Fig 4:**
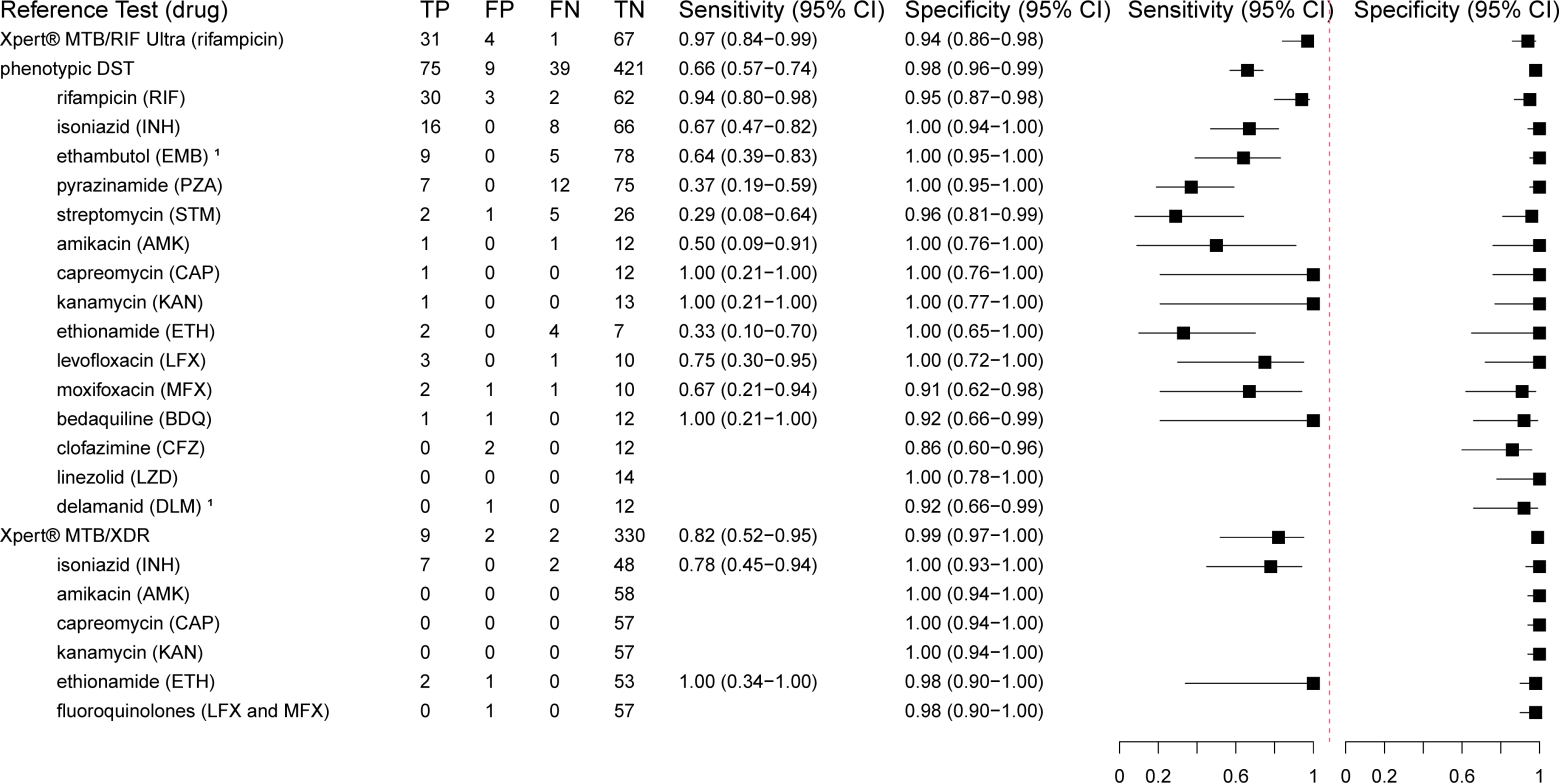
Diagnostic test accuracy of TBDR sequencing in detecting drug resistance compared to Xpert MTB/RIF Ultra, phenotypic DST, and Xpert MTB/XDR using decontaminated sputum samples. TP—true positive, FP—false positive, FN—false negative, TN—true negative, and 95% CI—95% confidence interval. ^1^Predicted TBDR drug susceptibility of 20 supplementary drug-resistant samples was compared to predicted susceptibility based on whole-genome sequencing.

We calculated the sensitivity and specificity of TBDR sequencing in detecting drug resistance on decontaminated sputum samples using Xpert MTB/RIF Ultra, phenotypic DST, and Xpert MTB/XDR (available only in South Africa) as the reference standard (Fig. 4). Compared to Xpert MTB/RIF Ultra, TBDR sequencing showed a sensitivity of 97% (95% CI 84–99%) and a specificity of 94% (95% CI 86–98%) for rifampicin. The sensitivity of TBDR sequencing compared to phenotypic DST across all drugs evaluated was 66% (95% CI 57–74%). For rifampicin, sensitivity was 94% (95% CI 80–98%) but 67% (95% CI 47–82%) for isoniazid and 64% (95% CI 39–83%) for ethambutol. TBDR sequencing did not detect variants associated with pyrazinamide resistance in 12 out of 19 samples phenotypically resistant to pyrazinamide. Compared to the Xpert MTB/XDR*,* TBDR sequencing showed an improved but uncertain sensitivity (82%, 95% CI 52–95%) when calculating the total sensitivity across all drugs evaluated, with a drug-specific sensitivity for isoniazid of 78 (CI: 45–94%) and 100% (CI: 0.34–100) for ethionamide. We did not find any cases of resistance for fluoroquinolones (levofloxacin, moxifloxacin), kanamycin, amikacin, and capreomycin in the samples sequenced and tested with Xpert MTB/XDR. The specificity of the TBDR assay was 92% or higher for all drugs and all reference tests used, except clofazimine. Test accuracy estimates calculated using the sequencing results of unprocessed samples are reported in Fig. S3A. A sensitivity analysis, excluding the supplementary drug-resistant samples, is reported in Fig. S3B, and TBDR sequencing is compared to a combined reference standard of all available DST results in Fig. S3A and C. Table S7 lists test accuracy estimates for total and first-line TB drugs compared to the combined reference standard, stratified by Xpert MTB/RIF Ultra bacterial load. Test accuracy estimates remained similar in all scenarios.

### Reproducibility of sequencing outcomes retested at a central laboratory

We calculated the percentage agreement between sequencing outcomes from the sites and those from the central laboratory in Switzerland for a subset of 20 paired processed and decontaminated samples (*n* = 40). Among the 40 samples, 15 were concordant failures, and 15 were concordant successes. Six samples that were successfully sequenced at the sites failed when retested at the central laboratory, while four samples that failed at the sites were successfully sequenced at the central laboratory (Table S8). The percentage agreement of sequencing in Southern Africa and Switzerland was 75% (30/40) with a Cohen’s kappa coefficient of 0.5, indicating moderate agreement of sequencing outcomes.

## DISCUSSION

We successfully implemented the TBDR assay at two clinical laboratories in Sub-Saharan Africa, obtaining sequencing results from unprocessed (native) and processed (decontaminated) sputum samples with a laboratory turnaround time of under 24 h. While decontaminated sputum samples showed higher success rates with fewer missed sequencing targets, unprocessed sputum samples also produced comprehensive drug susceptibility profiles, including second-line drugs. Although sequencing success rates varied between the two laboratories, the specificity of the TBDR assay was high in both settings.

The accuracy of tNGS in specialized and research settings has previously been reported (3–5). Here, we describe a prospective study reflecting the performance, implementation, and accuracy of tNGS in a routine diagnostic setting in high TB-burden countries. We compared the performance of TBDR sequencing in laboratories with varying levels of experience in NGS. We used standardized study protocols, consolidated laboratory procedures, and an automated analysis workflow to evaluate the TBDR test performance across different settings. The large number of drug-susceptible TB patients allowed for a robust evaluation of the test’s specificity.

Like previous diagnostic test accuracy studies evaluating tNGS assays, we had to exclude a considerable number of samples from our evaluation of diagnostic accuracy due to undetermined sequencing results (6, 7). We could not definitively attribute the discrepancy in sequencing success rates between the two sites to operator experience with the assay, batch-to-batch variabilities in the assay reagents, or differences in sputum sample quality. Retesting at the central laboratory showed discrepancies in sequencing outcomes compared to the ones from the sites, which suggests that operator experience or reagent differences may partially affect sequencing outcomes. Ideally, sequencing a set of control samples at both sites would have allowed us to better assess the impact of these factors. Additionally, Oxford Nanopore Diagnostics has designed its tNGS assay for use with decontaminated sputum, and we found that decontamination, which removes non-mycobacterial species and contaminants, improved sequencing success. However, any additional pre-processing would increase the complexity of testing, adding laboratory requirements that could limit the accessibility of tNGS in resource-limited settings.

The specificity of the TBDR assay in this study was high compared to other genotypic and phenotypic DST and comparable to the high specificity reported for tNGS assays (9). Sensitivity was variable compared to phenotypic DST, ranging from 33 (ethionamide) to 94% (rifampicin), and most pyrazinamide resistances (12/19) were missed. However, phenotypic DST is considered unreliable for pyrazinamide (19, 20), with estimates in 22 test accuracy studies ranging from 50 to 100% (9). The relatively low sensitivity in our study could be partially attributed to the small number of resistant cases, resulting in uncertain sensitivity estimates. Additionally, the TBDR assay relies on a curated catalogue of mutations and discards unknown variants, potentially contributing to the observed false negatives compared to culture-based phenotypic DST. However, a key advantage of tNGS is its flexibility: the mutation catalogue or the gene targets can be updated based on new evidence or setting-specific differences with minimal impact on available infrastructure and testing procedures. An updated version of the TBDR assay, AmPORE TB, which interprets mutations using the second edition of the WHO Catalogue of Mutations (2023) (17), performed equally well as other tNGS technologies in detecting drug-resistance associated mutations (21). We did not calculate predictive values since the prevalence of resistance in our study does not necessarily reflect the true prevalence in South African and Zambian populations. The missed resistances and lower sensitivity of the TBDR assay observed in this study limit the utility of this current version as a standalone diagnostic tool for select drugs. It may need to be used in conjunction with other diagnostic methods as part of a diagnostic algorithm to ensure comprehensive detection of drug-resistant TB.

Successful sequencing of decontaminated and unprocessed sputum samples at two sites with different capacities and levels of NGS experience demonstrates that TBDR sequencing is feasible in diverse settings. Despite the inherent complexity of targeted NGS, the standardized TBDR protocol facilitated its successful implementation at both sites. At the Zambian site, which had no prior experience with nanopore sequencing, the automated analysis pipeline mitigated a lack of bioinformatics capacity, illustrating how standardized protocols and bioinformatics automation can enable advanced sequencing technologies in diverse settings. TBDR sequencing showed flexibility and robustness when adapting it to the local context. While the assay is designed to process 22 samples per sequencing run for maximal cost-effectiveness, we achieved successful results with fewer samples from decontaminated and unprocessed sputum samples. Other studies have also demonstrated the rapidness and simplicity of nanopore library preparation and sequencing and the low cost and high portability of MinION sequencers compared to other technologies (21–23). This makes nanopore tNGS an attractive option for regional or district laboratories, aiming to expand comprehensive DST beyond national reference laboratories.

There are limitations to our study. For many samples, predominantly unprocessed samples, the paired sample was not sequenced, and these samples were excluded from the logistic regression. The excluded unprocessed samples had a higher success rate, potentially leading to an underestimation of the sequencing success rate of unprocessed samples and consequently overestimating the differences in sequencing outcomes between decontaminated and unprocessed samples. Moreover, the incidence of drug resistance in the cohort was low, resulting in uncertain sensitivity estimates, and the sensitivity of the TBDR assay could not be precisely determined for several drugs.

We encountered several logistic and technical issues, which underscore the difficulty in translating scientific innovations into scalable, effective solutions in clinical practice, particularly in resource-limited settings (24–26). A lack of local suppliers and long delivery times delayed sequencing. The shelf-life of reagents and their storage conditions need to be considered, especially in settings with low throughput. We experienced driver installation failures on sequencing laptops and software update issues, which required remote intervention from technical specialists. Despite the automation, the complexity of NGS workflows requires robust IT support and troubleshooting capabilities. Similarly, TBDR sequencing, as implemented in our study, requires manual library preparation. Minor differences in pipetting and reagent handling techniques by different operators may impact library amplification and ultimately influence sequencing success. This highlights the importance of continuous training and capacity building, unidirectional PCR infrastructures, or wet lab automation to ensure consistent and reproducible performance across laboratories. The higher failure rate observed in samples with low bacterial load may limit the clinical utility of TBDR sequencing for paucibacillary TB cases. However, we found that the test’s accuracy remained unaffected by bacterial load in our study, which suggests that enhancing the detection sensitivity, for example, by improving DNA extraction yields or optimizing target amplification, could substantially improve its performance.

In summary, targeted amplicon sequencing is a rapid and comprehensive genotypic DST method that produces drug resistance profiles for several TB drugs simultaneously, including novel second-line treatments, and can be used with unprocessed sputum samples. Opportunities for technical and operational improvements remain, but the TBDR assay promises to be a valuable addition to current DST methods, particularly to provide an initial result with a rapid turnaround time while phenotypic DST is ongoing, or in settings where other DST methods for second-line drugs are not available.

## Data Availability

TBDR tNGS data are available in the Sequence Read Archive of the National Center for Biotechnology Information as FASTQ files under study accession no. PRJNA1196171. Data and code used for analysis are available on https://github.com/tianacs/TBDR_implementation.

## SUPPLEMENTAL MATERIAL

Supplemental material jcm.01390-24-s0001.docx Tables S1 to S8; Figures S1 to S3.

ASM does not own the copyrights to Supplemental Material that may be linked to, or accessed through, an article. The authors have granted ASM a non-exclusive, world-wide license to publish the Supplemental Material files. Please contact the corresponding author directly for reuse.
