# Supplementary material for: Field evaluation of nanopore targeted next-generation sequencing to predict drug-resistant tuberculosis from native sputum in South Africa and Zambia
Source: J Clin Microbiol. 2025 Feb 12;63(3):e01390-24. doi: 10.1128/jcm.01390-24 (PMC11898686; doi:10.1128/jcm.01390-24)

**SUPPLEMENTARY MATERIAL**  *J Clin Microbiol*

**Field Evaluation of Nanopore Targeted Next-Generation Sequencing to Predict Drug-Resistant Tuberculosis from Native Sputum
in South Africa and Zambia**

Tiana C. Schwab ^1^, Lavania Joseph ^2^, Andrew Moono ^3^, Pauline C. Göller ^4^, Mamello Motsei ^2^, Guy Muula ^3^, Denise Evans ^5^, Stefan Neuenschwander ^6^, Gunar Günther ^7,8^, Carolyn Bolton ^3^, Peter M. Keller ^9^, Alban Ramette ^6^, Matthias Egger ^1,10,11^, Shaheed V. Omar ^2^, Lukas Fenner ^1^, on behalf of IeDEA Southern Africa (IeDEA-SA)

**1** Institute of Social and Preventive Medicine, University of Bern, Bern, Switzerland

**2** Centre for Tuberculosis, National & WHO Supranational TB Reference Laboratory, National Institute for Communicable Diseases, a division of the National Health Laboratory Services, Johannesburg, South Africa

**3** Centre for Infectious Disease Research in Zambia, Lusaka, Zambia

**4** Institute of Medical Microbiology, University of Zürich, Switzerland

**5** Health Economics and Epidemiology Research Office, Faculty of Health Sciences, University of the Witwatersrand, Johannesburg, South Africa

**6** Institute for Infectious Diseases, University of Bern, Bern, Switzerland

**7** Department of Pulmonology and Allergology, Inselspital, Bern University Hospital, Bern, Switzerland

**8** Department of Medical Science, Faculty of Health Sciences, University of Namibia, Windhoek, Namibia

**9** Clinical Bacteriology/Mycology, University Hospital Basel, Basel, Switzerland

**10** Centre for Infectious Disease Epidemiology & Research, School of Public Health &Family Medicine, University of Cape Town, Cape Town, South Africa

**11** Population Health Sciences, Bristol Medical School, University of Bristol, Bristol, UK

Supplementary Table S1: Targets amplified for the TBDR assay by drug compound, critical concentration used for phenotypic DST and drugs included in Xpert® MTB/XDR assay.

| **Drug** | **Gene targets** | **Phenotypic DST  (critical concentration, µg/mL)** | **Xpert® MTB/XDR** | **Whole-genome sequencing^1^** |
| --- | --- | --- | --- | --- |
| Isoniazid (INH) | *inhA, fabG1, katG* | 0.1 | X |  |
| Rifampicin (RIF) | *rpoB* | 0.5 |  |  |
| Ethambutol (EMB) | *embB, embA* | 5.0 |  | X |
| Pyrazinamide (PZA) | *pncA* | 100 |  |  |
| Streptomycin (STM) | *rpsL, rrs, gidB* | 1.0 |  |  |
| Amikacin (AMK) | *rrs* | 1.0 | X |  |
| Capreomycin (CAP) | *rrs, tlyA* | 2.5 | X |  |
| Kanamycin (KAN) | *rrs, eis* | 2.5 | X |  |
| Ethionamide (ETH) | *inhA* | 5.0 | X |  |
| Moxifloxacin (MFX) | *gyrA, gyrB* | 0.25 | X |  |
| Levofloxacin (LFX) | *gyrA, gyrB* | 1.0 | X |  |
| Bedaquiline (BDQ) | *Rv0678, atpE* | 1.0 |  |  |
| Clofazimine (CFZ) | *Rv0678* | 1.0 |  |  |
| Delamanid (DLM) | *ddn, fgd1* |  |  | X |
| Pretomanid (PMD) | *ddn, fbiA, fbiB, fbiC, fgd1* |  |  |  |
| Linezolid (LZD) | *rplC, rrl* | 1.0 |  |  |

^1^ Whole-genome sequencing was done using Illumina NextSeq 2000 (Illumina, San Diego, CA, USA) and mutations classified as resistance-conferring for the indicated drugs by the WHO Catalogue of Mutations, 2^nd^ edition (2023).

X denotes drugs included in Xpert® MTB/XDR assay or for which whole-genome sequencing was used as a reference standard.

**Supplementary Table S2**: TBDR assay validity. To ensure a sequencing run is valid, the analysis workflow assesses the coverage (number of reads) of each gene target (amplicon) in both the control and test samples. The following thresholds apply to each of the samples included in a sequencing run.

| **Sample** | **Threshold required for successful sequencing** |
| --- | --- |
| Test sample | *Hsp65* target successfully covered (median coverage 20x or higher)  AND/OR  15 or more DR targets successfully covered (median coverage 20x or higher)  OR  Internal control (IC) successfully covered (median coverage 20x or higher) |
| No template control | Less than 20x median coverage in fewer than three gene targets. |
| Positive control | 20x median coverage or higher in 22 or more gene targets. |

Supplementary Table S3: STARD 2015 checklist

|  | **Section & Topic** | **No** | **Item** | **Reported on page #** |
| --- | --- | --- | --- | --- |
|  |  |  |  |  |
|  | **TITLE OR ABSTRACT** |  |  |  |
|  |  | **1** | Identification as a study of diagnostic accuracy using at least one measure of accuracy  (such as sensitivity, specificity, predictive values, or AUC) | N/A |
|  | **ABSTRACT** |  |  |  |
|  |  | **2** | Structured summary of study design, methods, results, and conclusions  (for specific guidance, see STARD for Abstracts) | p.2 * JCM does not seem to have structured abstracts |
|  | **INTRODUCTION** |  |  |  |
|  |  | **3** | Scientific and clinical background, including the intended use and clinical role of the index test | p.4 |
|  |  | **4** | Study objectives and hypotheses | p.5 |
|  | **METHODS** |  |  |  |
|  | *Study design* | **5** | Whether data collection was planned before the index test and reference standard  were performed (prospective study) or after (retrospective study) | p.6 |
|  | *Participants* | **6** | Eligibility criteria | p.6 |
|  |  | **7** | On what basis potentially eligible participants were identified  (such as symptoms, results from previous tests, inclusion in registry) | p.6 |
|  |  | **8** | Where and when potentially eligible participants were identified (setting, location and dates) | p.6+23 |
|  |  | **9** | Whether participants formed a consecutive, random or convenience series | p.6 |
|  | *Test methods* | **10a** | Index test, in sufficient detail to allow replication | p.7 |
|  |  | **10b** | Reference standard, in sufficient detail to allow replication | p.7+8 |
|  |  | **11** | Rationale for choosing the reference standard (if alternatives exist) | p.7 |
|  |  | **12a** | Definition of and rationale for test positivity cut-offs or result categories  of the index test, distinguishing pre-specified from exploratory | p.9 |
|  |  | **12b** | Definition of and rationale for test positivity cut-offs or result categories  of the reference standard, distinguishing pre-specified from exploratory | p.8+30 |
|  |  | **13a** | Whether clinical information and reference standard results were available  to the performers/readers of the index test | NA |
|  |  | **13b** | Whether clinical information and index test results were available  to the assessors of the reference standard | NA |
|  | *Analysis* | **14** | Methods for estimating or comparing measures of diagnostic accuracy | p.9 |
|  |  | **15** | How indeterminate index test or reference standard results were handled | p.9 |
|  |  | **16** | How missing data on the index test and reference standard were handled | p.9 |
|  |  | **17** | Any analyses of variability in diagnostic accuracy, distinguishing pre-specified from exploratory | NA |
|  |  | **18** | Intended sample size and how it was determined | NA – Since this was primarily a field evaluation, a reasonable intended sample size was too uncertain to estimate |
|  | **RESULTS** |  |  |  |
|  | *Participants* | **19** | Flow of participants, using a diagram | p.26 |
|  |  | **20** | Baseline demographic and clinical characteristics of participants | p.24 |
|  |  | **21a** | Distribution of severity of disease in those with the target condition | NA |
|  |  | **21b** | Distribution of alternative diagnoses in those without the target condition | NA |
|  |  | **22** | Time interval and any clinical interventions between index test and reference standard | p.6 |
|  | *Test results* | **23** | Cross tabulation of the index test results (or their distribution)  by the results of the reference standard | p.29+38 |
|  |  | **24** | Estimates of diagnostic accuracy and their precision (such as 95% confidence intervals) | p.29+38 |
|  |  | **25** | Any adverse events from performing the index test or the reference standard | NA |
|  | **DISCUSSION** |  |  |  |
|  |  | **26** | Study limitations, including sources of potential bias, statistical uncertainty, and generalisability | p.15+18 |
|  |  | **27** | Implications for practice, including the intended use and clinical role of the index test | p.15-17 |
|  | **OTHER INFORMATION** |  |  |  |
|  |  | **28** | Registration number and name of registry | NA |
|  |  | **29** | Where the full study protocol can be accessed | NA |
|  |  | **30** | Sources of funding and other support; role of funders | p.19 |
|  |  |  |  |  |

NA – not applicable

Supplementary Table S4: Spoligotypes detected by TBDR sequencing in 173 decontaminated sputum samples. Lineage associated with spoligotype as in (Napier et al., 2023)

| **Lineage** | **Spoligotype** | **Zambia** | **South Africa** |
| --- | --- | --- | --- |
| Lineage 1 | EAI1-SOM |  | 1 |
|  | EAI5 |  | 1 |
|  | EAI6-BGD1 |  | 1 |
| Lineage 2 | Beijing |  | 28 |
| Lineage 3 | CAS | 1 |  |
|  | CAS1-Delhi |  | 3 |
|  | CAS1-Kili | 2 | 3 |
| Lineage 4 | Cameroon |  | 1 |
|  | H1 |  | 1 |
|  | H3 |  | 1 |
|  | LAM11-ZWE | 2 | 3 |
|  | LAM3 | 1 | 3 |
|  | LAM4 |  | 1 |
|  | LAM9 | 1 | 2 |
|  | S |  | 3 |
|  | T | 1 | 1 |
|  | T1 | 2 | 9 |
|  | T2 | 1 |  |
|  | T3 | 1 | 1 |
|  | X1 | 2 | 2 |
|  | X2 | 1 | 2 |
|  | X3 |  | 5 |
| Unknown | | 2 | 1 |
| No result ^1^ | | 59 | 24 |
| Total | | 76 | 97 |

^1^ Spoligotyping was unsuccessful because there was no coverage of the spoligotype target (n = 109), coverage was below the 20x coverage threshold (n = 3) or the spoligotype target was off target (n = 43).

Napier G, Couvin D, Refrégier G, Guyeux C, Meehan CJ, Sola C, et al. (2023). Comparison of in silico predicted *Mycobacterium tuberculosis* spoligotypes and lineages from whole genome sequencing data. *Sci Rep*. 13(1):11368.

Supplementary Table S5: Number of drug resistance-conferring mutations detected in 236 sequenced samples.

| **Drug** | **Variant** | **Zambia** | **South Africa** |
| --- | --- | --- | --- |
| Amikacin (AMK), capreomycin (CAP), kanamycin (KAN) | rrs.n.1401A>G |  | 1 |
| Bedaquiline (BDQ) & clofazimine (CFZ) | Rv0678.dropout |  | 1 |
|  | Rv0678.p.Glu49fs |  | 1 |
| Delamanid (DLM) & pretomanid (PMD) | fbiA.p.Leu125fs |  | 2^1^ |
| Ethambutol (EMB) | embB.p.Met306Ile | 2 | 3 |
|  | embB.p.Met306Val | 1 | 3 |
|  | embB.p.Gln497Arg |  | 2 |
|  | embB.p.Tyr319Ser |  | 1 |
| Ethionamide (ETH) & isoniazid (INH) | inhA.c.-777C>T | 1 | 3 |
|  | inhA.c.-154G>A |  | 1 |
|  | inhA.c.-770T>A |  | 1 |
| Isoniazid (INH) | katG.p.Ser315Thr | 4 | 9 |
|  | katG.p.Ser315Asn | 1 |  |
|  | katG.p.Ser315Arg |  | 1 |
| Levofloxacin (LFX) & moxifloxacin (MFX) | gyrA.p.Ala90Val |  | 2 |
|  | gyrA.p.Asp94Gly |  | 2 |
| Pyrazinamide (PZA) | pncA.p.His57Asp | 1 |  |
|  | pncA.p.Cys14Arg |  | 2 |
|  | pncA.p.Cys14Trp |  | 1 |
|  | pncA.p.Gly132fs |  | 1 |
|  | pncA.p.Gly97Asp |  | 1 |
|  | pncA.p.Leu172fs |  | 1 |
|  | pncA.p.Thr153fs |  | 1 |
| Rifampicin (RIF) | rpoB.p.Ser450Leu | 9^2^ | 18 |
|  | rpoB.p.His445Asp | 1 | 3 |
|  | rpoB.p.His445Tyr | 1 | 2 |
|  | rpoB.p.Asp435Val | 1 | 1 |
|  | rpoB.p.His445Leu | 1 |  |
|  | rpoB.p.Leu430Pro | 1 |  |
|  | rpoB.p.Leu452Pro |  | 2 |
|  | rpoB.p.Asp435Gly |  | 1 |
|  | rpoB.p.Gln432Lys |  | 1 |
|  | rpoB.p.Gln432_Phe433del |  | 1 |
|  | rpoB.p.His445Asn |  | 1 |
| Streptomycin (STM) | rpsL.p.Lys43Arg | 2 |  |
|  | rpsL.p.Lys88Arg | 1 | 1 |
|  | gid.p.Leu35fs | 1 |  |
|  | gid.p.Pro38fs | 1 |  |

^1^Mutation detected in one unprocessed sample but not in paired decontaminated sample.

^2^In two samples, mutation was detected in decontaminated samples but not in unprocessed.

Supplementary Table S6: Discrepancies in detected variants between paired unprocessed and decontaminated samples.

| **Sample** | **TB drug** | **Unprocessed** | **Decontaminated** | **Comments** |
| --- | --- | --- | --- | --- |
| ZM-1 | Rifampicin | rpoB.p.Leu430Pro | rpoB.p.Leu430Pro;  rpoB.p.Ser450Leu | Sample rifampicin-resistant by *Xpert® MTB/RIF Ultra* |
| SA-1 | Delamanid & pretomanid | fbiA.p.Leu125fs | - | No reference test available |
| SA-2 | Rifampicin | - | rpoB.p.Ser450Leu | Sample was rifampicin-susceptible by *Xpert® MTB/RIF Ultra* and phenotypic DST. |

Supplementary Table S7: Diagnostic test accuracy of TBDR sequencing using decontaminated samples compared to a combined reference standard. The combined reference standard was defined by combining the results from all available drug susceptibility test (Xpert® MTB/RIF Ultra, phenotypic DST, whole-genome sequencing, or Xpert® MTB/XDR). A sample was classified as drug-resistant if resistance was detected by any of these tests. Results are stratified by Xpert® MTB/RIF Ultra bacterial load.

| Sensitivity (95% Confidence Interval) | | | | |
| --- | --- | --- | --- | --- |
| **Drug** | **Total (n)** | **High (n)** | **Medium (n)** | **Low (n)** |
| Total | 0.68 (0.59–0.76) (838) | 0.68 (0.53–0.80) (369) | 0.63 (0.49–0.75) (306) | 0.74 (0.55–0.87) (163) |
| RIF | 0.94 (0.81–0.98) (103) | 0.94 (0.73–0.99) (50) | 0.92 (0.65–0.99) (34) | 1.00 (0.65–1.00) (19) |
| INH | 0.67 (0.47–0.82) (92) | 0.80 (0.49–0.94) (44) | 0.60 (0.31–0.83) (30) | 0.50 (0.15–0.85) (18) |
| EMB | 0.64 (0.39–0.83) (92) | 0.25 (0.05–0.70) (45) | 0.83 (0.43–0.97) (31) | 0.75 (0.30–0.95) (16) |
| PZA | 0.37 (0.19–0.59) (94) | 0.14 (0.02–0.51) (44) | 0.33 (0.12–0.64) (33) | 1.00 (0.44–1.00) (17) |
|  |  |  |  |  |
| Specificity (95% Confidence Interval) | | | | |
| **Drug** | **Total (n)** | **High (n)** | **Medium (n)** | **Low (n)** |
| Total | 0.99 (0.98–1.00) (838) | 1.00 (0.99–1.00) (369) | 0.98 (0.95–0.99) (306) | 0.99 (0.96–1.00) (163) |
| RIF | 0.99 (0.93–1.00) (103) | 1.00 (0.90–1.00) (50) | 0.95 (0.78–0.99) (34) | 1.00 (0.76–1.00) (19) |
| INH | 1.00 (0.95–1.00) (92) | 1.00 (0.90–1.00) (44) | 1.00 (0.84–1.00) (30) | 1.00 (0.78–1.00) (18) |
| EMB | 1.00 (0.95–1.00) (92) | 1.00 (0.91–1.00) (45) | 1.00 (0.87–1.00) (31) | 1.00 (0.76–1.00) (16) |
| PZA | 1.00 (0.95–1.00) (94) | 1.00 (0.91–1.00) (44) | 1.00 (0.86–1.00) (33) | 1.00 (0.78–1.00) (17) |

EMB, Ethambutol; INH, Isoniazid; PZA, Pyrazinamide; RIF, Rifampicin

Supplementary Table S8: Agreement of sequencing outcomes at the sites and retested with TBDR at a central laboratory (Institute of Infectious Diseases, University of Bern, Bern, Switzerland) for a subset of 20 paired processed and decontaminated samples (n=40).

| Sequencing outcomes at central laboratory | |  | Sequencing outcomes at local site | |
| --- | --- | --- | --- | --- |
|  | |  | Fail | Pass |
|  | Fail |  | 15 | 4 |
|  | Pass |  | 6 | 15 |

Supplementary Figure S1: Percentage of samples (n = 148) for which the respective gene target was successfully sequenced with a median coverage of 20x or higher. Internal control target (qc) is competitive; the presence of drug resistance gene targets or *hsp65* will prevent detection of qc target.


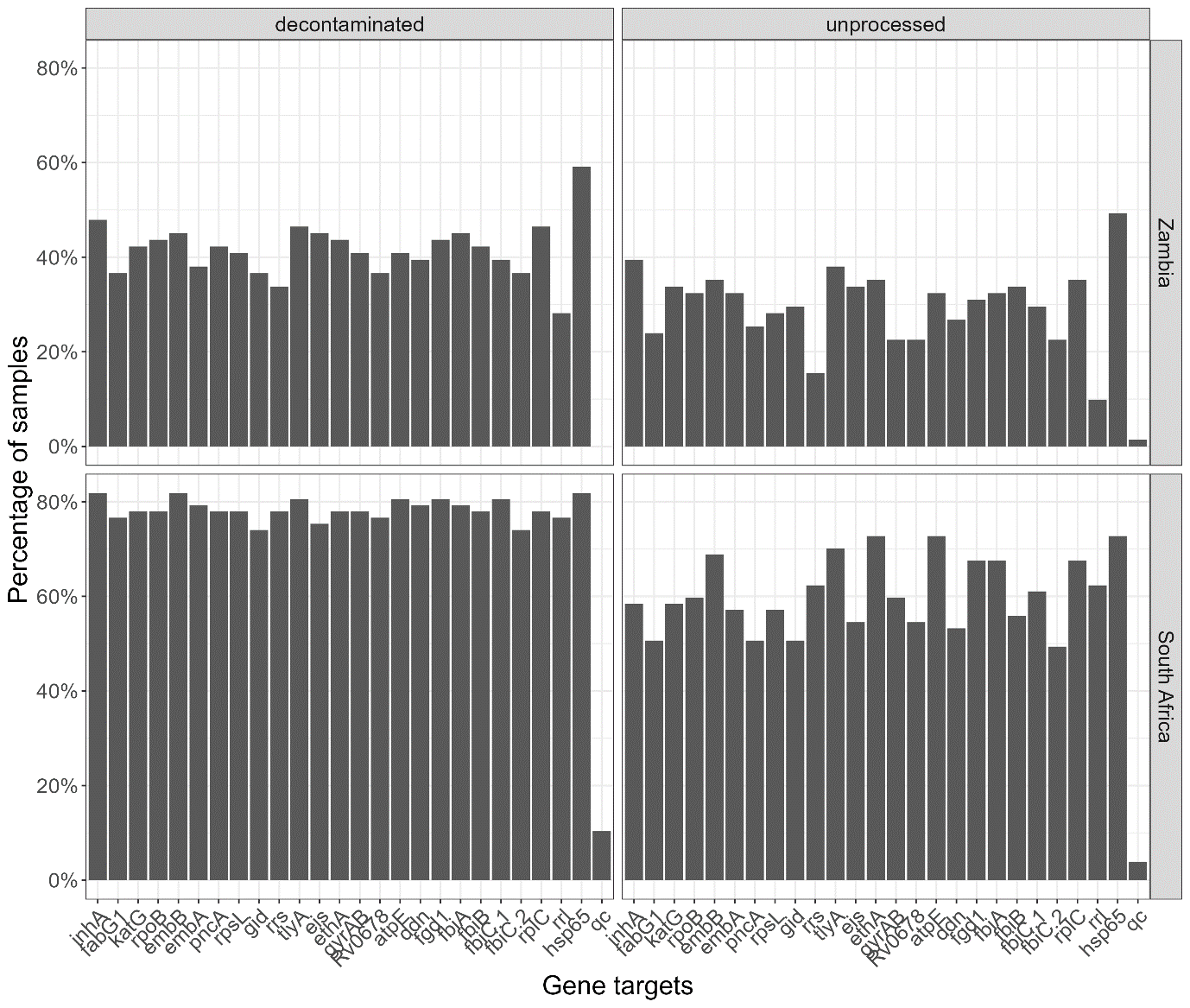


Supplementary Figure S2: Number of targets with insufficient coverage for drug resistance prediction per decontaminated or unprocessed sample, stratified by site and bacterial load (high/medium/low) as measured by Xpert® MTB/RIF Ultra (n = 148).


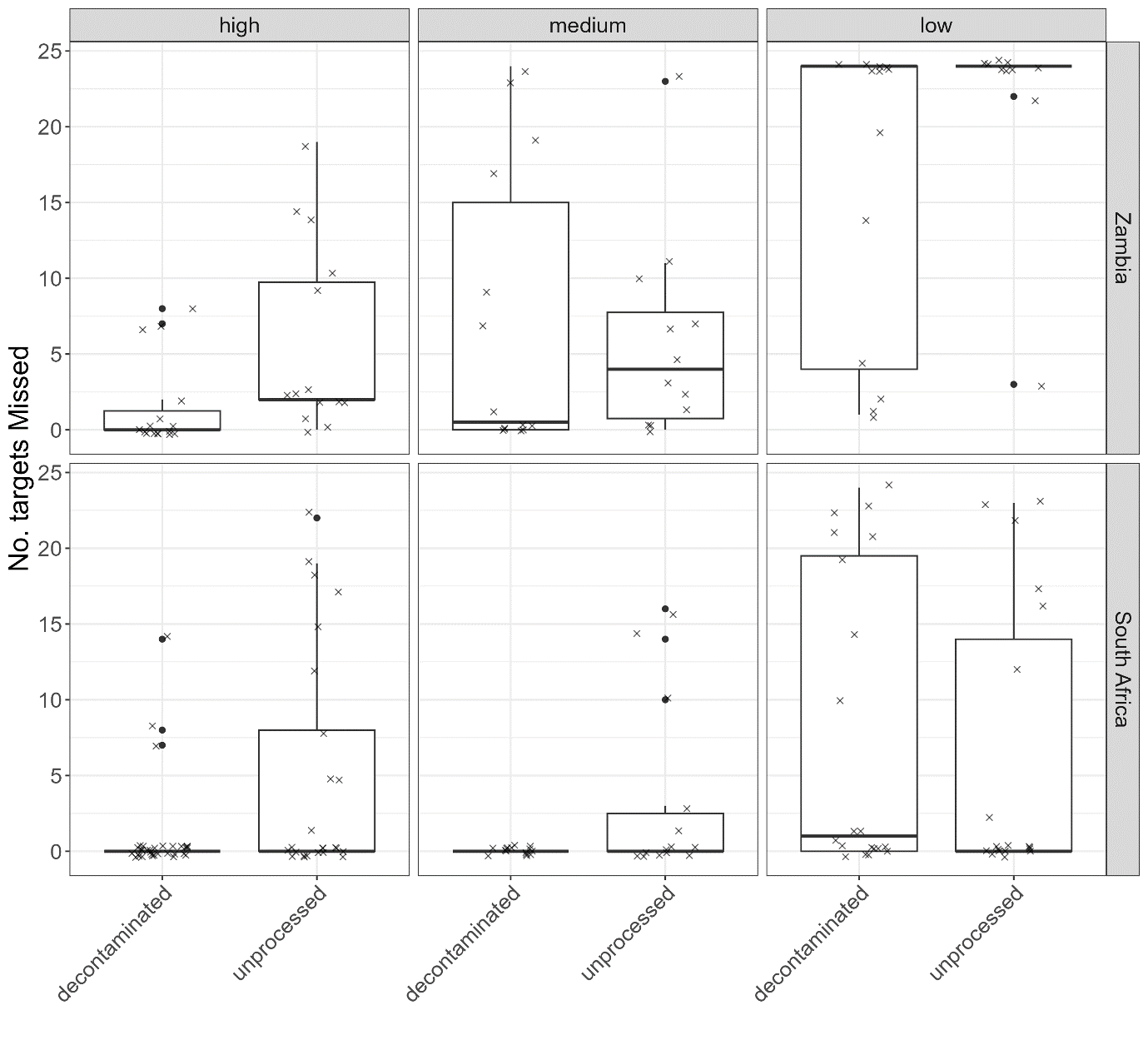


**Supplementary Figure S3:** Diagnostic test accuracy of TBDR sequencing compared to Xpert® MTB/RIF Ultra, phenotypic DST and Xpert® MTB/XDR using unprocessed sputum samples (A) or decontaminated samples excluding the supplementary set of drug-resistant samples (B). We compared the performance of TBDR sequencing using all decontaminated samples against a combined reference standard (C). The combined reference standard was defined by combining the results from all available drug susceptibility test (Xpert® MTB/RIF Ultra, phenotypic DST, whole-genome sequencing, or Xpert® MTB/XDR). A sample was classified as drug-resistant if resistance was detected by any of these tests.

TP – true positive, FP – false positive, FN – false negative, TN – true negative, 95% CI – 95% confidence interval.

**A**


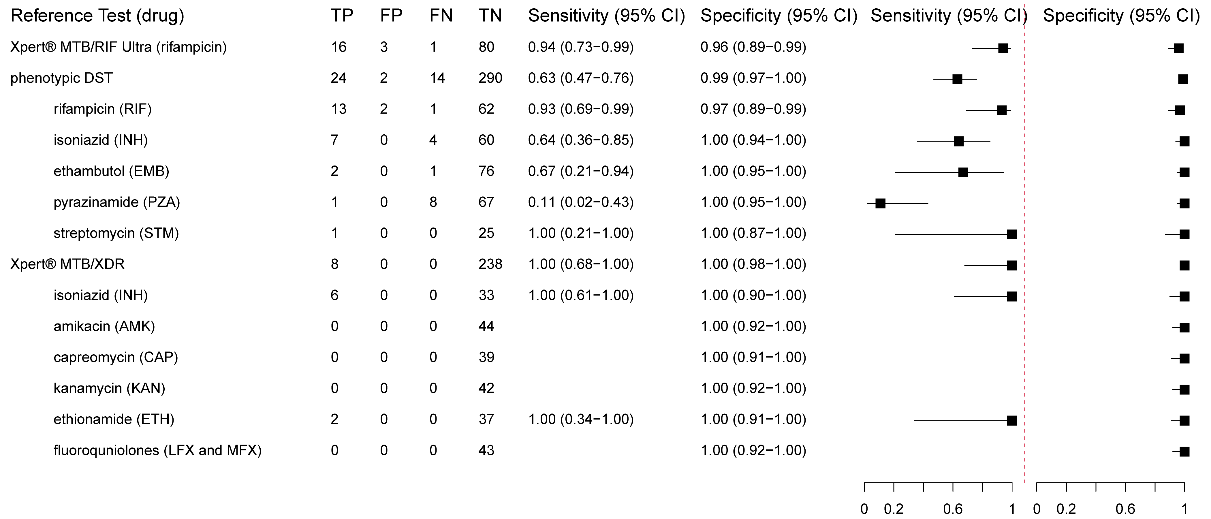


**B**


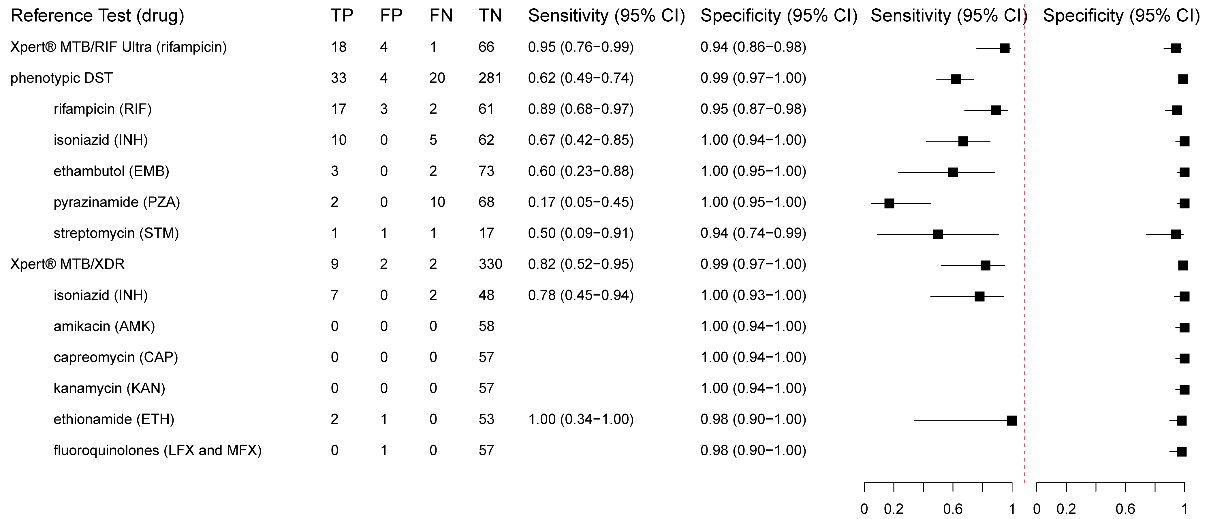


**C**


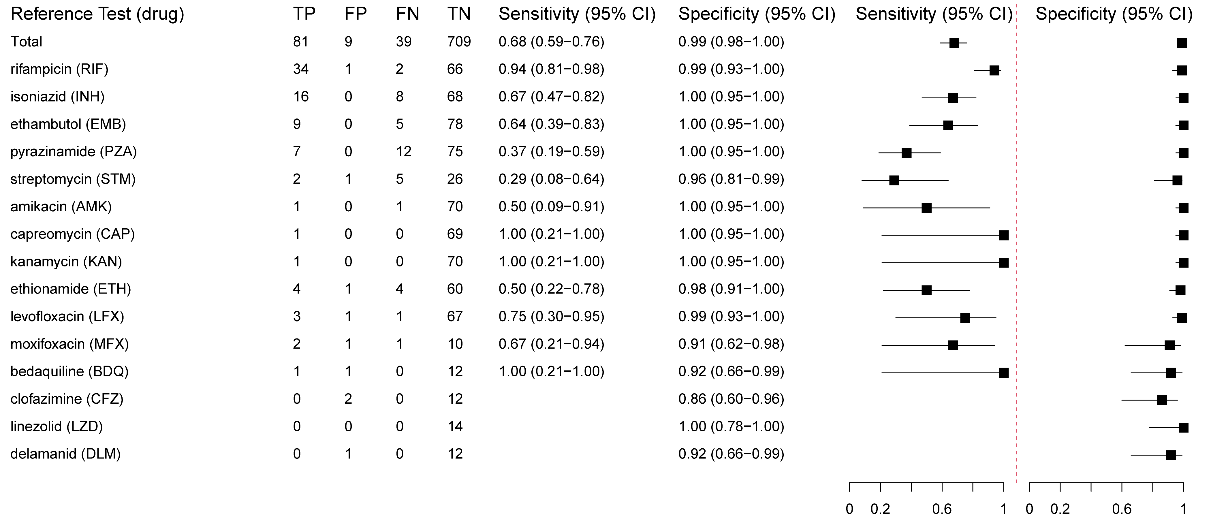

Supplement: Supplemental material — Tables S1 to S8; Figures S1 to S3. [file jcm.01390-24-s0001.docx]
